# Use of unstructured intervention in a community‐based mental health setting for the recovery of people with depression in Hong Kong: A qualitative assessment

**DOI:** 10.1111/hsc.13999

**Published:** 2022-09-04

**Authors:** Daniel W. L. Lai, Vincent W. P. Lee, Yong‐Xin Ruan

**Affiliations:** ^1^ Faculty of Social Sciences Hong Kong Baptist University Kowloon Tong Hong Kong; ^2^ Department of Social Work The Chinese University of Hong Kong Hong Kong City Hong Kong

**Keywords:** depression, psychosocial intervention, treatment outcome

## Abstract

This qualitative evaluation study examined the impact of unstructured intervention on people with depression in a community mental health centre in Hong Kong. In the intervention, service users initiated groups and designed group activities by themselves, with social workers as facilitators. Semi‐structured interviews were conducted with service users enrolled in unstructured intervention, their family members, and service providers in 2019. Overall, 32 participants were recruited through purposive sampling. The results revealed that peer interactions helped participants to feel accepted and understood. Participants also acquired experience with emotional coping strategies and practised their interpersonal skills, and by learning new skills from peers, they were able to rebuild their self‐esteem and improve their relationships with friends and family. To cater to clients' different needs and concerns, unstructured activities should be diversified. Family and social functioning should also be emphasised in the development of unstructured intervention because the generic skills and knowledge acquired from unstructured activities with peers could help to enhance family relationships, self‐confidence, and the ability to manage issues related to working and socialising.

**What is known about the topic?**

- Unstructured intervention gives autonomy to participants to determine the intervention process for dealing with mental health issues.
- Limited qualitative research has examined contributions of unstructured intervention in the recovery of depression of Chinese clients.

**What this paper adds**

- Unstructured intervention helps clients feel acceptance through peer interactions, enabling rebuilding of identity, and strengthening a sense of self‐worth for better social reintegration.
- Unstructured intervention distracts participants from unhealthy activities, facilitating more self‐control in their lives.
- Learning new generic skills from peer activities improved participants' social relationships.

## 1 INTRODUCTION

Depression is one of the most common mental disorders, affecting more than 264 million people worldwide (World Health Organization [WHO], 2020). Severe depression leads to suicide, causing over 800,000 lives annually (McLaughlin, 2011; WHO, 2020). Over the years, researchers and practitioners have been examining and applying various treatments and intervention approaches to better support the patients, as well as those under recovery. Currently, effective treatments for depression are mainly based on psychotherapy (Hollon et al., 2005). Moderate or severe depression is mainly treated by medicines, such as selective serotonin reuptake inhibitors and tricyclic antidepressants (Blanco et al., 2003). Yet, researchers (Lopez‐Montoyo et al., 2019; Wykes & Drake, 2012) have cautioned of the side effects of pharmacological treatment, such as excessive dependence and high relapse rates. On the other hand, the common types of psychotherapy include cognitive therapy, behavioural therapy, cognitive‐behavioural therapy, and psychosocial therapy. (Hollon et al., 2005). Although a combination of psychotherapy and pharmacotherapy could be a desirable treatment option (Lopez‐Montoyo et al., 2019), the usual intervention in primary care settings is mainly in the form of pharmacotherapy, while some patients prefer psychotherapy to pharmacological treatment. It is often challenging to introduce psychotherapy in primary care settings due to problems, such as the professionals' training, high costs or work overload, professionals' attitudes, and geographical and logistic difficulties (Castro et al., 2015; Rodgers et al., 2012).

In response to these challenges, the mental health practice communities in different parts of the world focus services on recovery‐oriented care frameworks, such as the Integrated Recovery‐oriented Model (Commonwealth of Australia, 2013; Frost, Tirupati, et al., 2017). Rehabilitation intervention should be tailored to individual needs. In terms of recovery, remediation, restoration, and reconnection are three crucial components (Frost, Turrell, et al., 2017), which accommodate the changing needs of patients during rehabilitation. This trend has drawn the attention of academics and practitioners to the unstructured intervention approach, as these approaches emphasise individual interests and the psychological implications of decision‐making processes elicited through patient‐driven group interaction. With the support and facilitation of the mental health workers, approaches to improve social support might be conducted in various ways, including peer support, skill‐building, group‐based activities, psychoeducation, psychotherapy, exercise, and links to community resources (Nagy & Moore, 2017). Participants could determine the central themes in terms of sequence and intensity without established rules (Lehman et al., 2015) and the frequency of intervention activities according to their needs (Wasilewski et al., 2017), while facilitators assist in participation and discussion (Al‐Yateem & Rossiter, 2017; Morriss et al., 2016). Due to its emphasis on developing clients' individual needs, interests, and aptitudes, unstructured intervention has gradually gained popularity (Brown et al., 2014; Kahl et al., 2020; Nguyen et al., 2019).

Yet, academic evidence about its applicability and suitability in supporting persons who are recovering from depression is relatively scant in the literature. Some research results had pointed out that the unstructured intervention approaches were usually applied in various ways, like peer support, skill‐building, group‐based activities, and information networks, could help to improve participants' mental health, social connectedness, self‐esteem, as well as lower their anxiety and stress levels (Al‐Yateem & Rossiter, 2017; Crossman et al., 2015; Pate et al., 2016; Subramanian et al., 2015). Some studies compared the impact and effectiveness of structured and unstructured intervention for treating people with mental health problems. Structured psychoeducation and training programmes were no more clinically effective than those of intensive unstructured intervention using peer support (Morriss et al., 2016; Subramanian et al., 2015), whereas unstructured group support for persons with bipolar disorder was more cost effective as clients had paid less for the unstructured services while achieving similar recovery results (Camacho et al., 2017).

International migration pattern and increase of cultural diversity in different societies would further justify the need to examine impact of mental health intervention on clients of different ethnic background. Previous research on psychosocial intervention with specific ethnic groups, such as Chinese, shows that structured interventions suit the socio‐cultural context and characteristics of the Chinese clients (Ng & Wong, 2018; Yin et al., 2020). Yet, the relatively limited studies on unstructured intervention in mental health have thus revealed the knowledge gap in the applicability, receptiveness, and relevance of unstructured intervention in a Chinese cultural context. This qualitative study aimed to understand the impact of unstructured intervention on the recovery of Chinese people with depression by evaluating the experiences and outcomes of an unstructured intervention programme in a community centre for mental wellness in Hong Kong.

## 2 MATERIALS AND METHODS

### 2.1 Study context

The study took place in a community centre for mental wellness, which provides district‐based community support and social rehabilitation services for people with previous mental health problems, people with suspected mental health problems, their families or caregivers, and residents in the serving community. This centre is one of the Integrative Community Centres for Mental Wellness (ICCMW), funded by the government and established under the official policy to establish one ICCMW for every 330,000 people. These centres are operated by NGOs in all the 18 administrative districts in Hong Kong with the overall mandate to provide services including individual counselling, outreach visits, occupational assessments and training, therapy groups, recreational activities, and community education programmes. Patients receiving mental health treatment in government mental health clinics are often referred to the ICCMW in the community where the clients reside. A case management approach is usually adopted by the centre so that the clients would receive relevant mental health intervention and support services from the professional team consisting mainly of social workers and related mental health practitioners.

The centre initiating the use of unstructured intervention is in the Southern District of the city. The practitioners of the centre initiated unstructured groups with three to five members after matching them with similar interests and backgrounds. They were exposed to opportunities to meet others and socialise with them. All clients with diagnosis of depression in the centre's membership were introduced to these unstructured peer groups. No other specific inclusion or exclusion criteria were adopted. Participation was voluntary and members were free to join or leave the groups on their own.

### 2.2 Conceptual framework

**FIGURE 1:**
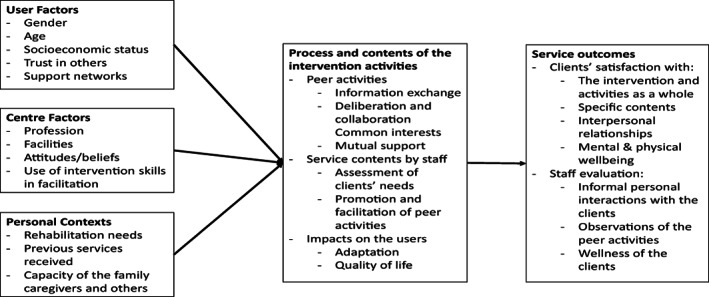
Conceptual framework for studying the perceived effectiveness of and satisfaction with unstructured intervention

To evaluate the experiences and outcomes of the unstructured intervention programme, this study adhered to an integrated evaluation framework adapted from the work process and service outcomes model proposed by Leon‐Carlyle et al. (2009). As indicated in Figure 1, various factors were conceptualised in relation to (1) perceived effectiveness of unstructured intervention and effects on service users, including how service users adapt to service modes; (2) interpersonal relationships; (3) reactions to new environments before and after participating in activities; (4) quality of life; (5) level of social integration; and (6) progress of recovery. These factors could be related to how the practitioners work with service users and the peer interactions for developing the content of the support programmes and activities promoted by unstructured intervention. These factors were expected to influence the outcomes of the unstructured intervention activities.

The design and establishment of the research questions, data collection methods, and data analyses were based on the predictive factors and the assessment areas contained in the evaluation framework, which accounts for the perspectives of service users, staff, and other various factors. The research questions were (a) What were the positive or negative experiences of unstructured intervention for different stakeholders? and (b) What were the outcomes of unstructured intervention on clients with depression?

### 2.3 Participants and Interviews

Between March and July 2019, in‐depth semi‐structured interviews were conducted with three target groups: (1) service users recovering from depression and enrolled in unstructured intervention activities, (2) caregivers or service users' family members, and (3) community centre staff members. Either in‐depth individual interviews or focus group interviews were conducted depending on the availability and preferences of the service users. Purposive sampling was adopted to identify respondents. Eligible participants were service users who were enrolled in the unstructured intervention programme, their family members or caregivers, and the staff members overseeing the unstructured intervention programme who could comment on the situations of their clients. To identify the participants, recommendations were first made by community centre staff members who had a thorough understanding of services users' availability and suitability. After communication between the centre staff and the research team over the suitability of the nominated participants, invitations were then distributed to the potential participants. To include participants representing their diverse contexts, considerations were given to the demographic diversity. Therefore, it was the intention of this study to include participants with different genders and age backgrounds. The principle of theoretical saturation was adopted in considering whether adequate interviews had been conducted. Research ethics approval was obtained from the Institutional Review Board of The Hong Kong Polytechnic University (HSEARS20190208001).

In total, 32 individuals were interviewed, including 24 service users enrolled in unstructured intervention activities, three caregivers or family members of those service users, and five community centre staff members. The interviews of service users had been coded and the research team continued to recruit service users for interviews until data saturation was reached. The relatively small number of caregivers/family members was due to the fact that some service users do not have a connection with any family members or caregivers in their recovery process. The staff members covered in our sample were mostly the staff involved in the service delivery. The service users were aged between 30 and 60. Among them, 21 were women and 3 were men. Due to privacy concerns, most service users did not choose to disclose much of their other personal background, such as specific details about their diagnosis, prognosis, or their family members. For caregivers or family members, two men and one woman were included and they were the husband, the son, and the wife of the service users respectively. Among the staff members interviewed, there were two caseworkers, one being a rehabilitation instructor, and two social workers responsible for this programme.

The focus of this study was to capture the perceived experiences of the participants. An interpretive research approach was adopted in line with the narrative approach (Anderson & Kirkpatrick, 2016), which was used to capture participants' life stories and experiences in specific situations or contexts (Riessman, 2008).

Semi‐structured interviews were conducted by a research assistant with a Master's degree in social work and the second and third authors who both have formal training in social work and human service areas. Three different types of interview questions were designed for these three target groups. For the service users, the questions asked included reasons for joining the programme; their feelings during the programme; and what changes they experienced after the intervention. For staff members, questions included the rationales for providing such intervention, concerns before the intervention, and the outcomes and difficulties encountered during the programme. For caregivers or family members, questions included their observations of the activities that the service users engaged in and changes in the service users' conditions. We were then able to identify and address the different experiences, feelings, and discourses among the stakeholders in the intervention programme. We also explored how the experiences of those participating in unstructured intervention activities were associated with different dimensions of change in service users' recovery from depression and psychosocial conditions, such as interpersonal relationships, social networks, acquisition of new skills and knowledge, self‐esteem, and self‐confidence.

The interviews were transcribed verbatim. The transcripts were reviewed on a case‐by‐case basis and coded line by line. Initial codes were generated and further aggregated into potential themes. The emerged codes and themes were further analysed according to their relevance to the research questions. The research team members took turns to independently review the coherence, accuracy, and distinctiveness of the themes emerged from the initial coding stages before the themes were consensually named to present the findings. The coding framework was presented in Table 1.

**TABLE 1:** Coding framework

Themes	Codes	Example quotes
Connectedness	Exchanging peers' supports	“I would ask other members not to overthink, especially for those who are in a bad mood or depressed. We just talk. They would also comfort me as well”
	Sustaining contact without interference of social workers	“In the end, my role was just to be informed. And they did more than just play badminton. They also went hiking and did other things”
	Reinforcing existing networks	“I do not have to face my son. I was often irritated by my son. Before visiting this centre, I often argued and fought with my son. But I could never win the fight. We often had arguments. Now that I often come to this centre, my relationship with him is better as well. We argue less … I get to talk to others and share my negative experiences with them at this centre. So, I am happier and more talkative now”
Optimism and hope for the future	Having positive thinking	“I had a new idea recently, probably because I joined this programme a few months ago … My past was terrible, and I had many failures … I hope I can have a brand‐new start. I have never had this thought before, perhaps because I have gained some positive energy from my time at the centre.”
	Gaining confidence towards recovery	“I find other members very positive. During the process, they help me. After getting to know them, I started coming to the centre more often. They motivated me to join activities more often … Now I have learned that I should never isolate myself in life. After seeing other members feeling happy, I want to be like them as well. I will take them as role models”
Building identity	Feeling accepted and understood	“They felt free and safe to share with one another because everyone in the centre comes from a similar background … the friends here in the centre understand one another because they all have had similar experiences”
	Finding a sense of self‐worth	“Now they can connect with other members who also value them and find them important. They may feel that there is value and a role for themselves. That feeling must matter. Perhaps the programme makes them understand their own value and roles”
Finding meaning in life	Finding values in relationship	“Before, I used to feel anxious. My mental status fluctuate a lot. I once spent 3 years at home. After I left the house for a bit, I hid myself again for more than a year … Then I joined the activities at the centre. I became happier, bolder, and more confident as I made more friends from “Best Friends Three Plus One”
	Rebuilding life	“I used to isolate myself. A social worker told me that I should open the door a little bit instead of shutting it completely … Yes, I can practise here now. This is a safe place to practise opening my heart. I dare not do so outside the centre. I keep my door shut completely outside the centre. But in the centre, I can learn and practise slowly”
Empowerment	Increasing sense of control	“I have fewer bad habits now. I spend less time gambling on horse racing and soccer … I have replaced my bad habits with something else, such as listening to songs”
	Providing a sense of belonging	“I feel happy coming to the centre … It is like returning to my parents' home. Every time I visit the centre, I do not want to go home”

## 3 RESULTS

In the intervention programme, social workers invited users with similar backgrounds to join in either activity including initiating a group activity by themselves and being tutors for other users in the programme. After joining at least three sessions of either activity, they were provided with subsidies to make plans with their counterparts and enjoy a leisure activity on their own either within or outside the centre. Staff members would intervene only in case of disputes to facilitate participants' active participation, usually in the early stage of the programme. The unstructured activities provided users autonomy to determine the contents with minimum intervention from the staff members. These strategies adopted were in alignment with some common characteristics of unstructured intervention in previous studies (Al‐Yateem & Rossiter, 2017; Lehman et al., 2015; Nagy & Moore, 2017).

**FIGURE 2:**
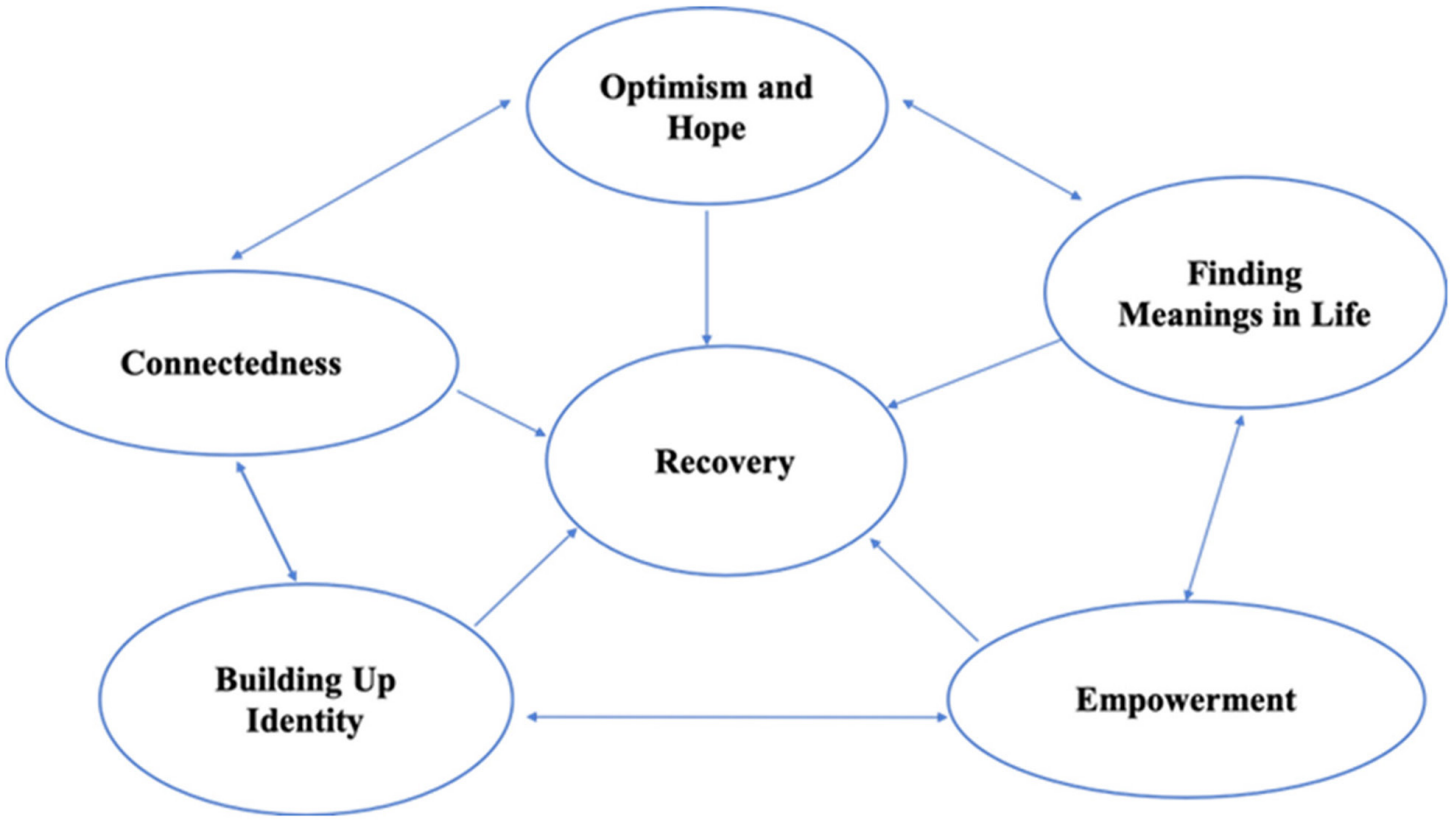
Five categories of “CHIME” recovery process

Anthony (1993, p. 21) defined personal recovery as “a deeply personal, unique process of changing one's attitudes, values, feelings, goals, skills and/or roles … a way of living [a] satisfying, hopeful and contributing life even with the limitations caused by illness.” Leamy et al. (2011, p. 448) indicated five recovery categories through the “CHIME” framework: (1) “connectedness”; (2) “hope and optimism about the future”; (3) “identity” (4) “meaning in life,” and (5) “empowerment.” The results of the interviews with the various stakeholders in this study were further interpreted with reference to the recovery categories of the CHIME framework (Figure 2). For the best interest and well‐being of people with mental health problems, recovery means gaining and retaining hope, understanding of one's abilities and disabilities, engagement in an active life, personal autonomy, social identity, meaning and purpose in life, and a positive sense of self. These factors would help to improve their growth and recovery from people with mental health problems. Each component of the CHIME framework does not stand alone in determining the extent of one's recovery, but it is interrelated to each other and mutually affected.

### 3.1 Nurturing connectedness between participants

“Connectedness” refers to themes related to social support, relationships, and community (Leamy et al., 2011). These connections are crucial to the progressive recovery of people with depression. The role of the unstructured intervention in enhancing connectedness with others was commonly mentioned by service users, their family members, and community centre staff. Seven staff members explained that the programme enabled users to build a social support network to cope with their emotions and feel supported: “I would ask other members not to overthink, especially for those who are in a bad mood or depressed. We just talk. They would also comfort me as well.”

A staff member shared the efforts in facilitating service users to participate in the activities:

It is very different from conducting group therapies. All the staff have to cooperate. It is never about one team or two who are responsible for the programme. It involves many case workers. They take up the roles as the initiators. They need to accompany the members to join the programme. They have invested a lot of time in it.

It is found that the service users maintained their networks by keeping in touch with their friends through social media, phone calls, or regular gatherings, which were done without any intervention or incentives from the programme. Participating in various activities also helped them cope with their negative emotions, which improved their relationships with their family members and friends outside the centre. As one service user said

I don't have to face my son. I was often irritated by my son. Before visiting this centre, I often argued and fought with my son. But I could never win the fight. We often had arguments. Now that I often come to this centre, my relationship with him is better as well. We argue less…I get to talk to others and share my negative experiences with them at this centre. So, I am happier and more talkative now.

The enhancement of connectedness through the unstructured intervention activities facilitated new friendships, exposed the participants to new activities, and improved their relationships with their friends and families, which positively affected their recovery.

### 3.2 Developing optimism and hope

“Optimism and hope for the future” refers to themes related to belief in and motivation for recovery, change, and success and to developing aspirations, positive thinking, and relationships that inspire hope (Leamy et al., 2011). During the interviews, service users reported an increase in their self‐efficacy, which strengthened their belief in their own ability and their potential to change. For instance, one service user expressed a positive perception of life and the future:

My past was terrible, and I had many failures … I hope I can have a brand‐new start. I have never had this thought before, perhaps because I have gained some positive energy from my time at the centre.

In addition, stronger optimism and hope could be attributed to the stronger sense of achievement the service users gained by assisting others:

I have learned how to cook and the steps involved in cooking … Before, I often felt that I was useless. Now I feel that I am useful. I have a sense of achievement … I feel happy after helping others.

Participants also reported that interacting with other service users who were in a more advanced stage of recovery reinforced their self‐efficacy towards recovery because they realised that their situations were not unique. As one service user reported,

I find other members very positive… After getting to know them, I started coming to the centre more often. They motivated me to join activities more often … Now I have learned that I should never isolate myself in life. After seeing other members feel happy, I want to be like them as well. I see them as role models.

Similar to previous findings identified in quantitative studies (Chaudhry et al., 2009; Travis et al., 2010; Wasilewski et al., 2017), our findings further illustrate the benefits of unstructured intervention. Service users gained a sense of achievement by helping others during the unstructured activities, which enabled them to transform their anxiety and negative self‐image into confidence and optimism. Recognition and encouragement from peers appeared to be more influential than those from staff members and played a vital role in further strengthening their self‐perception.

### 3.3 Building identity to overcome stigma

Building “identity” involves overcoming the stigma of mental health issues and rebuilding a positive self‐perception (Leamy et al., 2011), which is the basis of positive mental health. Five interviewees reported that the intervention enabled them to safely share their experiences of living with mental health issues, thereby lessening the stigma attached to people recovering from mental health issues. They recalled their experiences of not being accepted or understood by others. Both the staff members and service users explained that the unstructured intervention and the new social support networks they acquired provided a safe space to share their experiences of having people with mental health problems while still feeling accepted and understood. As one staff member said

I have heard many times from the members that it was nice for them to be able to share and talk in the centre because they might face discrimination outside. They felt free and safe to share with one another because everyone in the centre comes from a similar background … They felt like they found their partners in recovery.

Numerous participants echoed what the staff member said about participants' willingness to share things about themselves at the centre. Specifically, service users felt more comfortable communicating with their peers. As one service user said

We are all recovering from mental health problems. We understand that others must have experienced some tragic events in their lives. Now that we gather together, we feel happier. We are able to understand each other's pain.

Participants explained that they felt valued because of encouragement from others after completing intervention activities. The connections and group identity they formed during intervention further helped them to rebuild their self‐identity. A staff member also echoed this view:

Now they can connect with other members who also value them and find them important. They may feel that there is value and a role for themselves… Perhaps the programme makes them understand their own values and roles.

By participating in the unstructured intervention activities, the service users effectively managed to overcome the stigma attached to mental health issues and were able to rebuild positive self‐perception. Unlike some of their friends and even family members who did not experience depression themselves, their peers at the centre offered a suitable and comfortable platform for them to express themselves and be understood.

### 3.4 Finding meaning in life

Finding “meaning in life” refers to finding meaning in one's experiences with mental health problems, in one's own life, and in the quality of life, determining one's own social roles and goals, and rebuilding one's own life (Leamy et al., 2011). Nine interviewees reported increased quality of life. Some reported feeling better emotionally and having greater confidence. As one service user said,

Before, I used to feel anxious. My mental status fluctuate a lot. I once spent 3 years at home … Then I joined the activities at the centre. I became happier, bolder, and more confident.

The service users' families also recognised the positive effects of the unstructured activities on their emotions and recovery progress, as revealed by an interviewee's testimonial on the improved relationship between her and her daughter:

My daughter wrote a letter to the centre and told the staff that I had changed a lot … She said that after I came to the centre, it helped me a lot, because even they, as my kids, could not help me much… She also claimed that I used to be suicidal but that I have become more positive and empathetic to my family.

Furthermore, the activities and hobbies provided through intervention helped the participants to practise mindfulness and regulate their emotions. They felt less isolated after casually chatting with others and acquiring new knowledge. As one service user said:

I used to isolate myself. A social worker told me that I should open the door a little bit instead of shutting it completely … Yes, I can practise here now. This is a safe place to practise opening my heart.

### 3.5 Empowerment through programme activities

“Empowerment” refers to acquiring and focusing on responsibility, strength, and control in life (Leamy et al., 2011). Five service users described how the experiences and skills they first acquired during the unstructured intervention activities reshaped their perceptions regarding responsibility, strength, and control in life. Acquiring new skills and completing tasks reinforced participants' self‐confidence, enabling them to focus on their strengths and quit unhealthy habits. For example, a service user shared

I spend less time gambling on horse racing and soccer. I have gambled on horse racing for 47 years… Before, I used to go to the horse race course a lot. In the last 3 to 4 months, I have been there only once. I have replaced my bad habits with something else, such as listening to songs.

Some interviewees described the centre as their second home because it provided a safe place for them to bond with others who shared similar experiences. It is evident that a sense of belonging towards the centre had effectively empowered them. As one service user reported

I feel happy coming to the centre, especially because I live alone … Because I live nearby, it is easier for me to come to this centre. It is like returning to my parents' home. Every time I visit the centre, I do not want to go home.

## 4 DISCUSSION

Unstructured intervention helped service users to expand their social support networks, which further supported them to cope with their emotions. The service users were able to maintain the social networks they formed without any intervention from the staff as well as improving their relationships with their family members. They also expressed a sense of belonging to the community centre, which was considered a “second home” or “another shelter.” These results are consistent with those of several other studies (Chaudhry et al., 2009; Diefenbeck et al., 2017; DuBenske et al., 2014; Markowitz, 2015; Travis et al., 2010).

The unstructured intervention helped the service users to expand their knowledge and interests and enhance their self‐efficacy and self‐esteem; this outcome is consistent with the results of Beauchamp et al. (2005). Several participants reported an increased sense of achievement after participating in activities such as cooking or making art. Similar to Bremer et al.'s (2019) study indicating the improvement in self‐esteem among people with mental health problems through interpatient activities and mutual help, this study showed that the sense of achievement promoted by the unstructured interventions had improved the service users' self‐efficacy.

The intervention activities and the supportive environment of the centre empowered service users by enhancing their awareness of their strengths, enriching their daily lives, and reducing their involvement in unhealthy activities. These helped them to acquire a greater sense of control of their lives. These findings are consistent with those of Chambers et al. (2015), who described the positive effects of the recovery approach on the sense of control and autonomy among people with depression. The unstructured intervention also enabled participants to express themselves and build relationships, thereby enhancing their competence and self‐confidence.

The interviewees highlighted the efforts made by the staff members, who played a key role in enhancing the well‐being of people with depression. Their consistent reminders, warm invitations, and companionship initially caught the attention of the participants. In addition, the frequency with which the staff members interacted with clients influenced the clients' intervention experiences. Those who continually engaged in the intervention activities and built social support networks often described their interactions with other members in positive terms, whereas those who rarely did so were more likely to report experiences involving disputes or negative emotions. These findings are consistent with those of relevant studies which indicate that longer term participants were more likely to perceive unstructured activities as being beneficial and effective (Markowitz, 2015; Rebar & Taylor, 2017; Yip, 2018).

## 5 CONCLUSIONS AND IMPLICATIONS

This study found that the intervention facilitated recovery progress by enhancing social integration, expanding participants' knowledge and interest, enhancing self‐esteem and self‐efficacy, and improving interpersonal relationships. The activities and content of the unstructured intervention programmes should be diversified to cater to participants' personal needs and interests. When developing unstructured intervention programmes, service providers should consider the interests, preferences, needs, and concerns of different service users and motivate users to consider different types of programme activities. Service users' characteristics, socioeconomic status, and recovery progress should also be considered. By doing so, service providers can include more service users in unstructured intervention programmes. In addition to providing service users with sufficient autonomy to plan activities, each unstructured group should be occasionally followed up by a social worker, like the findings of research by Brown et al. (2014) and Lauzier‐Jobin and Houle (2022). These social workers would not necessarily need to assume full responsibility for decision‐making for the service users; rather, they should resemble mentors who assist the groups with their daily activities and provide them with advice if needed to ensure that the unstructured activities are sustainable. Service providers should help clients set clear objectives and targets to provide “expectation management,” which can help clients to consider the potential benefits of active participation and more clearly understand their own roles in the social circle.

The targeted, focused activities, and programme content that facilitate mental health recovery and social function could be effectively incorporated into the content of unstructured intervention. One possible approach is to strengthen service users' social capital by having them participate in unstructured intervention activities, from which more opportunities could be generated. Both social and instrumental needs should be considered. For example, several participants explained that their relationships with their family members had improved because they spent less time at home and experienced fewer conflicts. By obtaining new skills and knowledge and engaging in social gatherings, participants could divert their negative emotions to other activities. Therefore, unstructured intervention activities improve service users' relationships with their family members by providing them with more exposure to social networks and additional opportunities to acquire new knowledge and skills.

This study revealed that peer interactions in the unstructured activities helped participants recovering from depression to feel accepted and understood, which enabled them to strengthen their self‐worth and self‐identity. Participants also acquired emotional coping strategies and practised their interpersonal skills, which helped them to rebuild their self‐esteem and improve their relationships with friends and family. Nevertheless, this study has several limitations. First, this study has only covered the clientele of one ICCMW in Hong Kong. Yet, there are 23 more such centres and other mental health facilities across Hong Kong, so we believe that there may also be other unstructured intervention programmes. A larger sample of participants enrolled in the unstructured intervention programmes in Hong Kong would have made the study more representative. Second, only clients who had previous experience with depression were enrolled in the unstructured intervention programme, so we were unable to identify the effectiveness of such intervention on clients with different types of mental health issues. Third, we were unable to collect more detailed demographic data from the participants, so we cannot take their family background, educational attainment, and employment status into account. Future studies should use a sample of clients from more community centres to examine the diversity between different service settings and the effectiveness of unstructured intervention on clients with different backgrounds and mental health issues. In addition, further research about this intervention on Chinese clients in a cross‐cultural context, such as in western societies, is recommended.

As shown in this and previous studies, the role of practitioners in facilitating the unstructured activities is crucial for advising on the programme direction and maintaining cohesions between the group members. Although the participants in unstructured intervention should enjoy the highest degree of autonomy in running the activities, future studies and unstructured intervention should consider the “broker” or “facilitator” role of the practitioners in providing support and guidance in clients' recovery process.

## AUTHOR CONTRIBUTIONS

Daniel W. L. Lai and Vincent W. P. Lee conceptualised and designed this research study and paper. Vincent W. P. Lee and Yong‐Xin Ruan conducted data collection. All three authors involved in data analysis, interpretation, and writing the manuscript. All authors contributed to revisions and agreed on the final version submitted.

## FUNDING INFORMATION

At the time of this research study, all three authors were employed at the Hong Kong Polytechnic University with research supported by an anonymous funder operating the programme in this paper (Grant Number: P18‐0297) (Fu Hong Society).

## CONFLICT OF INTEREST

The authors declare no conflict of interest.

## Data Availability

Research data are not shared.
